# Optimized Roasting Conditions of Germinated Wheat for a Novel Cereal Beverage and Its Sensory Properties

**DOI:** 10.3390/foods11030481

**Published:** 2022-02-07

**Authors:** Thinzar Aung, Bo Ram Kim, Mi Jeong Kim

**Affiliations:** 1 Department of Food and Nutrition, Changwon National University, Changwon 51140, Korea; junothinzar88@gmail.com; 2 Interdisciplinary Program in Senior Human Ecology, Changwon National University, Changwon 51140, Korea; kinj56@changwon.ac.kr

**Keywords:** germinated wheat beverage, gamma-aminobutyric acid, total phenolic content, total flavonoid content, antioxidant capacity, response surface methodology

## Abstract

The objective of this study was to investigate the possibility of using germinated wheat as a nutritionally improved novel cereal beverage. To enhance the health-related functionality of a germinated wheat beverage (GWB), the roasting time and temperature of germinated wheat were optimized using a central composite design and response surface methodology. The optimum roasting conditions were determined as roasting temperature of 180 °C and roasting time of 44.56 min, resulting in maximum total flavonoid content (0.74 mg CE/g), total phenolic content (1.95 mg GE/g), 2,2-diphnyl-1-picrylhydrazyl (DPPH) radical scavenging activity (5.10 μM TE/g), Trolox equivalent antioxidant capacity (9.45 mM TE/g), and *γ*-aminobutyric acid content (2.25 mg/g). The germinated wheat roasted with optimum conditions was prepared in two types of GWB (hot and cold), and the sensory characteristics were tested by consumers (*n* = 102). The cold GWB showed relatively high preferences compared to hot GWB in appearance, odor, taste, and overall acceptabilities. In the intensity results of the sensory properties of GWB, the cold GWB tended to have stronger browning, grain odor, and nutty taste than the hot GWB. Conclusively, this study showed that optimizing the roasting conditions of germinated wheat could achieve desirable sensory properties and consumer acceptance while improving the health-related functionality of GWB.

## 1. Introduction

Wheat (*Triticum aestivum* L.) known as one of the world’s most important cereal crops, is widely utilized in the production of flour, malt, beer, and various end-uses such as bread, biscuits, noodle, pasta, and snacks [1,2]. In addition to serving as a staple cereal by providing calories from its carbohydrates and proteins, wheat contains bioactive compounds that are associated with various health benefits, including reduced risk of cardiovascular disease, cancer, and type II diabetes [3]. Therefore, the enrichment of bioactive compounds in wheat-related products has become a major focus of food product development.

Among the methods employed to fortify nutritional value, germination is one of the most promising ways to enhance bioactive compounds such as folate, *γ*-aminobutyric acid (GABA), tocopherols, vitamins, and flavonoids in cereal grains by activating related enzymes [3,4]. The germination process induces enzyme activation and antioxidant synthesis to protect the plant from oxidative damage [2]. For example, germination synchronously increases the levels of phenolic compounds such as *p*-coumaric, *p*-hydroxybenzoic, and syringic acids in Polish wheat [1]. Another study reported that germination can enhance the free and bound phenolic content, as well as increase antioxidant activity [5].

The numerous studies reported the improved nutritional quality of food products (bread, noodles, and crackers) formulated with germinated wheat flour, suggesting utilization of germination to enhance health benefits in grains [2,6,7]. Although the addition of germinated flours can enhance the nutritional value of products such as bread, noodles, and crackers, it can also affect the product’s technological properties, such as weakening the gluten network and decreasing water absorption by dough, resulting in sticky, gummy crumb, and poor texture in baked products [2,6,7]. Thus, suitable end-products using germinated grains might be suitable for beverage since the gluten network and texture are not important quality factors for beverages [8]. Moreover, grain-based beverages is gaining popularity in Asian countries, including South Korea and Japan, due to their unique aroma, taste, and health-promoting benefits [9]. However, no study has reported the development of germinated wheat tea and its health-related functionality.

The grain beverage manufacturing process requires roasting to create its unique flavor profile [10,11]. In addition, roasting can improve the consumer acceptability of beverage formulations by eliminating ethanoic and acetic acids and removing undesirable volatiles formed during germination [8]. However, roasting also produces negative effects, such as the participation of germination-derived reducing sugars and free amino acids in the Maillard reaction [12]. Thus, the selection of optimal roasting conditions is important to obtain a desirable germinated wheat beverage (GWB). Among potential parameters for optimization, time and temperature are reportedly the most important conditions during the roasting process of grains [13]. Response surface methodology (RSM) is a reliable and applicable tool used to optimize complicated research conditions that generate massive amounts of data. RSM employs three major steps: design of the experiment, statistical analysis, and optimization of the variables [14]. Studies have reported the application of RSM using central composite design (CCD) to optimize the roasting conditions of various products, such as coffee and hazelnuts [15,16]. 

The present study has optimized the roasting temperature and time conditions to enhance the health-related properties of a novel GWB using RSM. Furthermore, this study aimed to understand the quality characteristics of GWB prepared using the optimized roasted wheat. In addition, the quality attributes of GWB prepared as hot and cold beverages evaluated consumer acceptance and the intensity of the quality characteristics. Thus, the study findings could provide insights that will help improve cereal-based beverages prepared with germinated wheat to ensure optimum functionality and consumer acceptance.

## 2. Materials and Methods

### 2.1. Raw Materials and Chemicals

The Anzunbaengi wheat cultivar grown in Jinju, South Korea, was used to prepare GWB in this study. Gamma-Aminobutyric acid (GABA), catechin, gallic acid, 2,2-diphnyl-1-picrylhydrazyl (DPPH), potassium persulfate, trichloroacetic acid, potassium persulfate, aluminium chloride, and sodium hypochlorite were purchased from Sigma-Aldrich (St. Louis, MO, USA). Sodium hydroxide, methanol, and phenol were obtained from Dae Jung Co., Ltd. (Seoul, South Korea). 

### 2.2. Preparation of Roasted Germinated Wheat

After soaking the wheat in water (1:5 *w*/*v*) at room temperature (RT; 25 °C ± 2 °C) for 12 h, the wheat was germinated in an incubator at 17.6 °C for 46.18 h in accordance with previously described germination conditions [17]. The germinated wheat was then dried under hot air at 45 °C for 16 h. The dried germinated wheat was steamed at 220 °C for 10 min, and then placed in an incubator at 50 °C for 16 h according to the method described by Wu, et al. [18]. Subsequently, the germinated wheat was roasted under various temperature and time conditions using an MK-300 roaster (JC Company, Seoul, Korea). 

The roasting conditions for the germinated wheat were optimized using CCD in RSM. The two independent variables were set at five temperature (131.716, 140, 160, 180, and 188.284 °C) and time (1.716, 10, 30, 50, and 58 min) levels (Table 1). 

To obtain precise data, the analysis was replicated three times, and the resultant average data were used for each of the 13 experimental runs (Table 2). 

The quadratic equation for the dependent variables is shown as follows:

$$Y_{n}={\;\beta }_{0}+{\;\beta }_{0}+{\;\beta }_{1\;}X_{1\;}+{\;\beta }_{12}X_{1\;}X_{2\;}+\beta_{11}X_{1}^{2}{}_{\;}+\beta_{22}X_{2}^{2}{}_{\;}$$

where Y is the dependent variable representing browning index (BI), total flavonoid content (TFC), total phenolic content (TPC), DPPH radical scavenging activity (DPPH), Trolox equivalent antioxidant capacity (TEAC), and GABA content; β_0_ is a constant; β_1_ and β_2_ are linear coefficients; β_12_ is an interaction coefficient; β_11_ and β_22_ are quadratic coefficients; and X_1_ and X_2_ are the independent variables temperature and time, respectively.

### 2.3. Analysis of Responses

#### 2.3.1. Browning Index

The chromaticity characteristics (L*, a*, and b* values) of the roasted wheat samples were measured using a Capsure-RM200 spectrocolorimeter (X-Rite, Inc., Grand Rapids, MI, USA). BI was calculated using the following equations [19]:

$$X=\left(a^{*}+1.75{\;L}^{*}\right)/\left(5.645{\;L}^{*}+a^{*}-0.3012{\;b}^{*}\right)$$

BI=[100 (X−0.31)]/0.172

where L* represents the lightness value, a* represents the redness value, and b* represents the yellowness value. 

#### 2.3.2. Total Flavonoid Content

Roasted germinated wheat was extracted with 80% EtOH (1:5, *w*/*v*) in a shaking incubator at 100× *g* rpm for 3 h at 65 °C. After centrifugation at 4000× *g* rpm for 10 min, the supernatant was filtered through a 0.45 μm filter. Subsequently, the roasted wheat extract was stored at −20 °C until further analysis. 

TFC of the roasted wheat extract was measured using the spectrophotometric method described by Kim, Kwak, and Kim (2018). Briefly, 100 mL extract was mixed with 1.25 mL distilled water plus 75 mL 5% sodium nitrite and incubated for 6 min, after which 150 µL 10% aluminum chloride was added to the mixture. After reacting for 5 min, 0.5 mL 1 M sodium hydroxide was added to the mixture and the absorbance was measured at 510 nm using an EMC-11D-V spectrophotometer, (EMCLAB Instruments, Duisburg, Germany). Catechin hydrate was used as a standard, and the results were expressed in mg CE/g sample.

#### 2.3.3. Total Phenolic Content

TPC of the roasted wheat extract was determined using the method described by Gujral, et al. [20]. Briefly, 20 µL extract was mixed with 1.5 mL 20% sodium carbonate plus 500 μL 10% Folin–Ciocalteu reagent and reacted in the dark at RT for 2 h. Subsequently, the absorbance of the sample was measured at 765 nm using the EMC-11D-V spectrophotometer (EMCLAB Instruments, Dusiburg, Germany). Gallic acid was used as a standard, and the results were expressed in mg GE/g sample. 

#### 2.3.4. DPPH Radical Scavenging Activity

The antioxidant potential, i.e., DPPH radical scavenging activity, of the roasted wheat extract was measured following the method described by Wong, et al. [21]. Briefly, 50 mL extract was combined with 1950 μL 0.1 mM DPPH and reacted in the dark at RT for 30 min. The absorbance of the sample was determined at 515 nm using the EMC-11D-V spectrophotometer (EMCLAB Instruments, Dusiburg, Germany). The results were calculated based on the Trolox standard curve and values were expressed in μM TE/g sample.

#### 2.3.5. Trolox Equivalent Antioxidant Capacity

TEAC of the roasted wheat extract was analyzed using the spectrophotometric method described by Simsek and El [22]. A mixture of 7.4 mM 2,2′-azino-bis(3-ethylbenzothiazoline-6-sulfonic acid) (ABTS) and 2.6 mM potassium persulfate was incubated in the dark at RT for 16 h. The ABTS mixture was diluted with sufficient 100% MeOH to reach a value of 0.7 when absorbance was measured at 734 nm. Subsequently, 2980 μL diluted ABTS solution was mixed with 20 μL extract and reacted for 7 min at RT. The absorbance of the sample was measured at 734 nm using the EMC-11D-V spectrophotometer (EMCLAB Instruments, Dusiburg, Germany). The results were reported in mM TE/g sample based on the Trolox standard curve. 

#### 2.3.6. *γ*-Aminobutyric Acid Content

Prior to analysis of GABA content, roasted wheat samples were extracted according to the method described by Sharma, et al. [23]. Briefly, 1 g sample was mixed with 4 mL 8% trichloroacetic acid, followed by centrifugation at 3000× *g* rpm for 20 min. The supernatant was passed through 0.45 μM filter paper and stored at −20 °C until analysis. Subsequently, 100 μL extract was mixed with 0.2 mL 0.2 M borate buffer, 1 mL 6% phenol reagent, and 7.5% sodium hypochlorite in a water bath at 100 °C for 10 min. After cooling the mixture in ice water for 5 min, the absorbance of the sample was measured at 630 nm using the EMC-11D-V spectrophotometer (EMCLAB Instruments, Dusiburg, Germany). GABA was used as a standard, and the results were expressed in mg/100 g sample. 

### 2.4. Sensory Evaluation

Upon optimizing the roasting conditions, hot and cold GWB was prepared to determine consumer preference and intensity of the sensory characteristics. For hot GWB, germinated wheat roasted under optimized conditions was ground into powder, and tea bags were prepared containing 0.8, 2, and 4 g wheat powder. According to our market survey, commercial tea bag generally contains approximately 2 g per each bag, thus the amount of tea for hot infusion was set as 2 g. Each tea bag containing 0.8, 2, and 4 g wheat powder was infused in 100 mL boiling water for 25 min. After removing the tea bags, hot GWBs with different levels of GWB (0.8, 2, and 4 g) were obtained and then labeled as Hot GWB_1, Hot GWB_2, and Hot GWB_3, respectively. For the preparation of cold GWB, germinated wheats roasted under optimized condition (25, 50, and 75 g) was placed in containers filled with 1 L water, and then boiled for 30 min based on the method of Kim et al. [24]. After cooling the sample at RT for 10 min, the GWB was stored at 4 °C for further sensory evaluation. The cold GWBs prepared with 25, 50, 75 g of roasting germinated wheat were labeled as Cold GWB_1, Cold GWB_2, and Cold GWB_3.

Consumer preference and the intensity of the sensory attributes for GWB were evaluated by consumers (*n* = 102) at Changwon National University. All sensory evaluations were conducted in the sensory lab using a protocol approved by the Institutional Review Board of Changwon National University (7001066-202002-HR-004). First, consumer preference was measured using a 9-point hedonic scale (1 = dislike extremely; 9 = like extremely). Consumers were also asked to evaluate the intensity of GWB characteristics (brownness, grain odor, sweet odor, grass odor, nutty taste, sweet taste, bitter taste, and aftertaste) on a 5-point category scale (1 = too weak; 5 = too strong) according to the previous method with a slight modification on the nature of sample [24]. Samples of GWB were individually presented in white paper cups marked with three-digit codes, presented in random order. All sensory evaluations were conducted in individual booths. Bottled water was provided for mouth rinsing between sample evaluations. The panelists had a 1 min break between samples to minimize sensory fatigue.

### 2.5. Statistical Analysis

Design Expert Software version 11 (Stat-Ease Inc., Minneapolis, MN, USA) was used to analyze the experimental data to obtain best-fit model equations and response plots for BI, TFC, TPC, DPPH, TEAC, and GABA. The roasting temperature and time combination that generated the highest overall desirability were selected as the optimum roasting conditions. To validate the predicted optimized roasting conditions, the germinated wheat was roasted with the optimum temperature and time identified using CCD in RSM, and analyzed for the selected responses. The absolute residual error (%) was calculated using the predicted and actual data using the following equation [17]:

Absolute residual error(%)=(Actual value−Predicted value/Actual value)×100

All experiments were carried out in triplicate, and analysis of variance (ANOVA) was used to determine differences among samples using XLSTAT software (Addinsoft, Paris, France). The Duncan multiple comparison test was performed to determine significant differences at *p* < 0.05 among sample means.

## 3. Results and Discussion

### 3.1. Roasting Time-Temperature Impact on BI

The variations in BI, TFC, TPC, DPPH, TEAC, and GABA response values with respect to their independent variables are shown in Table 3. Color is an important indicator of flavor quality during roasting but is also an important visual characteristic of beverages. 

**Figure 1:**
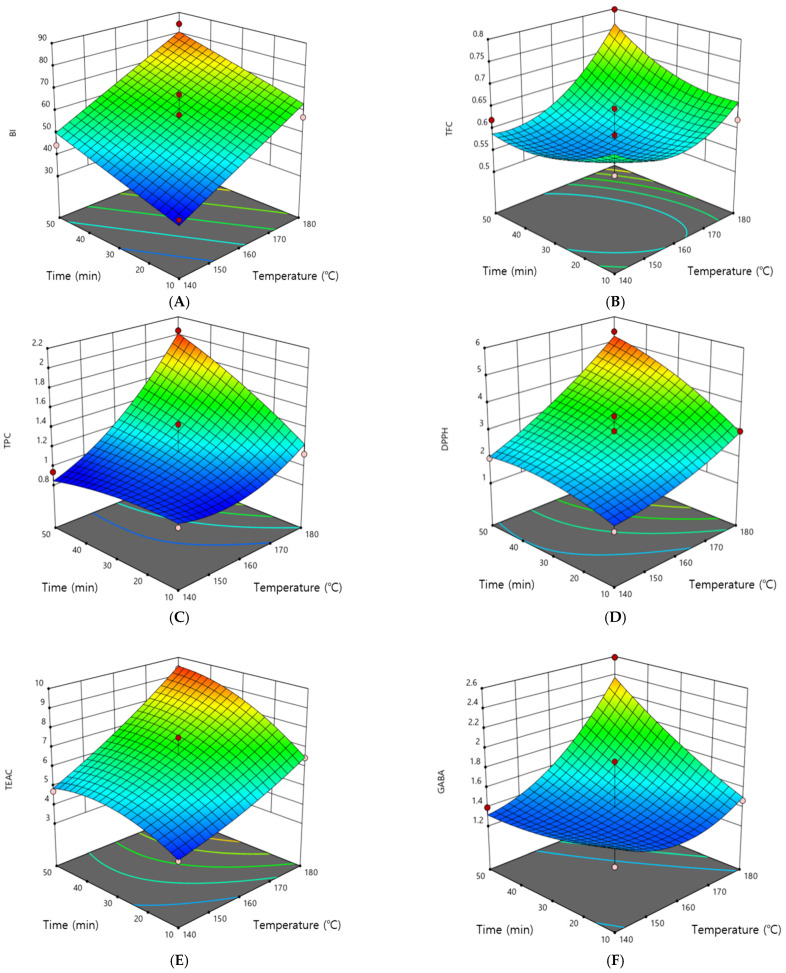
Three-dimensional response surface plots for BI, TFC, TPC, DPPH, TEAC, and GABA. (**A**) BI, browning index; (**B**) TFC, total flavonoid content (mg CE/g); (**C**) TPC, total phenolic content (mg GE/g); (**D**) DPPH, 2,2-diphenyl-1-picrylhydrazyl radical scavenging activity (μM TE/g); (**E**) TEAC, Trolox equivalent antioxidant capacity (mM TE/g); (**F**) GABA, gamma-amino butyric acid (mg/g).

BI values at different roasting time and temperature conditions ranged from 35.20–84.48, indicating a significant difference among the roasting treatments (*p* < 0.001). As shown in the three-dimensional response surface plots (Figure 1A), increased BI values were observed at higher temperatures and longer roasting times. The lowest BI value (35.20 ± 1.24) was determined when wheat was roasted at 140 °C for 10 min, while the highest BI value (84.48 ± 1.80) was obtained when wheat was roasted at the highest temperature (188.2 °C) for 30 min.

A similar darkening reaction has been reported in roasted chickpeas and barley flour at temperatures above 120 °C [13,25]. Darkening during roasting suggested that the reducing sugars and amino acids led to a non-enzymatic browning reaction, i.e., the Maillard reaction, during heat treatment [26]. Generally, this reaction is accelerated at higher temperatures and is related to flavor development in food products [27]. Thakur et al. reported that optimum BI of cereal based beverages using roasted maize was 79 [28]. Therefore, the BI value was set within the appropriate range to obtain favorable color development for further optimization of the roasting conditions. 

### 3.2. Roasting Time-Temperature Impact on Bioactive Compounds

Roasting time and temperature had a significant effect on the bioactive compounds (TFC and TPC) in the germinated wheat extract (Table 3). A gradual increase in TFC values was observed at higher temperatures and longer roasting times, indicating a significant difference among the roasting treatments (*p* < 0.001) (Figure 1B). Although the TFC value decreased slightly when germinated wheat was roasted at the highest temperature (188 °C) for 30 min, the highest TFC value (0.80 ± 0.04 mg CE/g) was observed when germinated wheat was roasted at 180 °C for 50 min. These results concurred with the observations by Park and Lee [29], who reported increased TFC values in lignin of omija fruit extract at higher roasting temperatures. These authors suggested that this phenomenon was related to the destruction of internal tissues during roasting, followed by the release of extractable phenols. 

A similar trend was also identified in TPC of the roasted wheat extract, revealing increased TPC values at higher roasting temperatures for longer periods (Table 3, Figure 1C). The TPC value significantly increased from 0.93 ± 0.07 mg GE/g when wheat was roasted at 160 °C for 1.7 min to 2.05 ± 0.10 mg GE/g when wheat was roasted at 180 °C for 50 min (*p* < 0.001). The TPC value decreased slightly (2.04 ± 0.11 mg GE/g) when germinated wheat was roasted at the highest temperature (188 °C) for 30 min (*p* > 0.001). Similar results demonstrating an increase in TPC values in the flours of grains and seeds at higher roasting temperatures have been reported in several studies [13,29,30]. Bound phenolic and flavonoid compounds are believed to be released during roasting due to destruction of the cell structure [30]. However, oxidation and thermal degradation of phenolic compounds also leads to increased production of extractable phenolics upon heating [13,31]. According to a previous report, wheat grain mainly contains ferulic and *p*-coumaric acids, which are generally found in conjugated forms bound to arabinoxylans and other cell wall polysaccharides [32]. These acids are generally released after hydrolysis during thermal treatment and then degraded to several types of simple phenolics [33].

### 3.3. Roasting Time-Temperature Impact on Antioxidant Capacity

In this study, the antioxidant capacity of the roasted wheat extracts was investigated by analyzing DPPH and TEAC (Table 3). Generally, the antioxidant capacity of the sample was related to its phenolic and flavonoid content. Hence, a similar trend was observed among bioactive compounds and antioxidant capacity during roasting (Figure 1). The DPPH radical scavenging capacity values ranged from 1.30 ± 0.45–5.41 ± 0.50 μM TE/g in germinated wheat extract when wheat was roasted at 131.7–188.2 °C for 10–58 min. The highest DPPH value (5.41 ± 0.50 μM TE/g) was obtained when wheat was roasted at 180 °C for 50 min, while the lowest DPPH value (1.30 ± 0.45 μM TE/g) was obtained when germinated wheat was roasted at 140 °C for 10 min. These outcomes are in fair agreement with the incremental increases in TPC and TFC values at higher roasting temperatures and a slight decrease in these values at the highest temperature, suggesting a relationship between TPC, TFC, and DPPH radical scavenging capacity. Gallegos-Infante, Rocha-Guzman, Gonzalez-Laredo and Pulido-Alonso [33] reported a similar result, hypothesizing that the increase in DPPH radical scavenging activity was attributable to the Maillard reaction due to the formation of thermally induced products that led to increased antioxidant potential during heating.

TEAC is a measurement of antioxidant potential used in the quality assessment of several food products, especially tea and beverages. The TEAC values increased significantly at higher roasting temperatures (*p <* 0.001) (Table 4). The highest TEAC value (9.76 ± 0.82 mM TE/g) was observed when germinated wheat was roasted at the highest temperature (188 °C) for 30 min, while the lowest TEAC value (3.94 ± 0.46 mM TE/g) was found when wheat was roasted at 140 °C for 10 min. Kocadağlı and Gökmen [34] reported that TEAC values increased with roasting time to a certain extent, and then decreased over a longer period of roasting, which concurred with the results of the current study. Moreover, an increasing trend in TEAC values was observed with increasing roasting temperature, which was consistent with some studies [35,36]. Similarly, these authors hypothesized that higher antioxidant capacity during high-temperature roasting may be attributable to biodegradation of bioactive compounds and generation of other products with antioxidant properties by the Maillard reaction.

### 3.4. Roasting Time-Temperature Impact on GABA Content

In this study, the GABA content of the germinated wheat extract increased to some extent with increasing roasting temperature, and then decreased slightly at higher temperatures (Figure 1F). The GABA values of the roasted wheat extract ranged from 1.12 ± 0.12–2.59 ± 0.09 mg/g, indicating significant differences among the roasting treatments (*p* < 0.001) (Table 4). GABA is important in the human diet to prevent neurological disorders and its levels are enhanced during the germination of cereals, including wheat, due to decarboxylation of L-glutamate [37]. The GABA values of roasted wheat extract increased with increasing temperature to some extent, and then decreased when wheat was roasted at the highest temperature (188 °C) for 30 min (2.08 ± 0.03 mg/g). Kim, Han, Lim, and Cho [8] suggested that the reduction in GABA values may be due to thermal decomposition of amino acids during high-temperature roasting. Therefore, optimized roasting conditions are necessary to obtain the highest GABA content under favorable conditions. 

### 3.5. Optimization of Roasting Time-Temperature by RSM

RSM is often used to determine optimal processing conditions in the food industry [17]. In this study, CCD was applied to determine the optimum roasting conditions for germinated wheat to develop a novel GWB. The optimization process involves the estimation of coefficient values, prediction of response values, and checking the acceptability of the model [15]. The important parameters, including the regression coefficient (R^2^), adjusted R^2^, lack of fit (*p*-value), and *p*-value (model), were analyzed using ANOVA, and are presented in Table 4. Lower probability values (*p* < 0.05), indicating insignificant lack of fit, higher R^2^ values (close to 1), and adjusted R^2^ values close to R^2^ values are indicators of the goodness of fit of the model [14]. In the current study, the linear model indicated all responses were significant in terms of low *p*-values. Higher R^2^ values were observed for all responses, with 0.8631 for BI, 0.7306 for TFC, 0.9190 for TPC, 0.9637 for DPPH, 0.9709 for TEAC, and 0.7499 for GABA. Additionally, all adjusted R^2^ values were close to R^2^ values. Insignificant lack of fit (*p*-value) and significant *p*-values were also found in BI, TPC, DPPH, TEAC, and GABA at different levels of significance (*p* < 0.05, *p* < 0.01, *p* < 0.001) (Table 4). The above data indicated that each response had good reliability and could be assumed to apply to the optimization process. Subsequently, optimization of the roasting conditions was performed to obtain the highest desirability by setting maximum values for TFC, TPC, DPPH, TEAC, and GABA, with BI set within the range of appropriate values. The optimized roasting conditions with the highest desirability index (0.861) were predicted to be 180 °C for 44.56 min.

### 3.6. Validation of Optimal Roasting Conditions

The predicted response values at the optimized roasting conditions were validated by performing a set of additional experiments and statistical assessment. Process validation of the optimal roasting conditions was performed by comparing the predicted response values with experimental values under optimized conditions using absolute residual error values (Table 5). 

The predicted values of for BI, TFC, TPC, DPPH, TEAC, and GABA were 78.73, 0.74 mg CE/g, 1.95 mg GE/g, 5.10 μM TE/g, 9.45 mM TE/g, and 2.25 mg/g, respectively. The experimental values for these factors obtained under optimal roasting conditions were 73.54 ± 1.80, 0.72 ± 0.03 mg CE/g, 1.96 ± 0.06 mg GE/g, 4.66 ± 0.18, 10.20 ± 1.23 mM TE/g, and 2.46 ± 0.14 mg/g, respectively. The absolute error value of TPC was less than 1%, observing 1.95 mg GE/g and 1.96 ± 0.06 mg GE/g for the predicted and experimental values. The predicted values for BI, TFC, and DPPH were slightly higher than the experimental values, whereas the experimental values for TEAC and GABA were higher than the predicted values. According to the data, the predicted and experimental values showed an error of less than 10%, confirming the accuracy of the model and indicating agreement between experimentally determined and statistically predicted response values.

### 3.7. Consumer Evaluation

The acceptability of hot and cold GWB prepared with the optimized roasted wheat was first evaluated by panelists who assessed four different sensory attributes (appearance, odor, taste, and overall acceptability) using a 9-point hedonic scale (Table 6). 

All four attributes in cold GWBs were preferred by panelists compared to hot GWBs, which may have been due to differences in the amount of roasted wheat powder and brewing methods used for the two preparations. According to a recent report [38], the effect of brewing temperature on consumer acceptability is pronounced, with consumers showing greater preference for cold tea beverages than hot GWBs. For hot GWBs, the amount of roasted wheat powder in the infusion exerted a significant effect on the sensory parameters (*p* < 0.001). Panelists assigned the highest appearance, taste, and overall acceptability ratings to hot GWB_2 (2 g/100 mL) among hot GWB samples, while the highest preferred odor was assigned to hot GWB_1 (0.8 g/100 mL). Regarding cold beverages, panelists preferred cold GWB_2 (50 g/1000 mL), which received the highest sensory attribute ratings among all samples (6.52 ± 1.18 for appearance, 6.54 ± 1.30 for odor, 6.62 ± 1.47 for taste, and 6.54 ± 1.41 for overall acceptability), indicating slight to moderate enjoyment of the beverage. 

**Figure 2:**
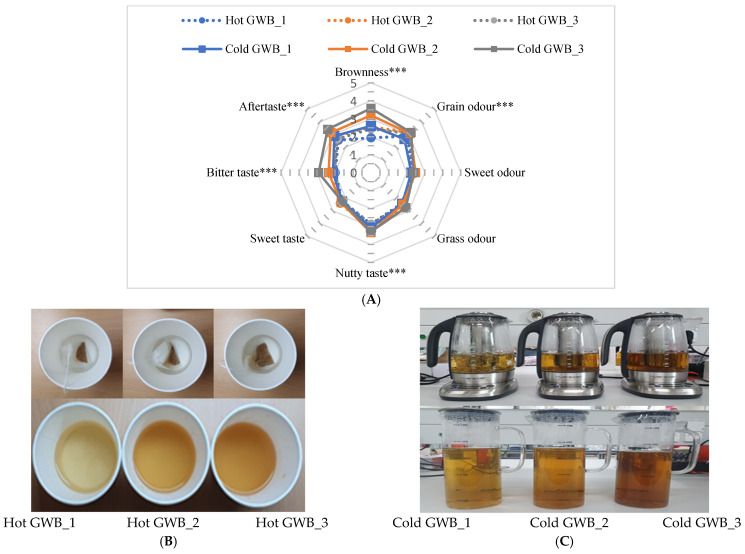
Spider chart for the intensities of sensory characteristics of germinated wheat beverage (GWB) prepared as (**A**) hot tea and cold beverage. Pictures of GWB prepared as (**B**) hot tea and (**C**) cold beverage. Hot GWB_1, Hot GWB_2, and Hot GWB_3 represent GWB prepared as hot tea at 0.8, 2, and 4 g/100 mL, respectively. Cold GWB_1, Cold GWB_2, and Cold GWB_3 represent GWB prepared as a cold beverage at 25, 50, and 75 g/1000 mL, respectively. *** indicates a significant difference at *p* < 0.001.

Intensity scores of hot and cold GWB prepared with the optimized roasted wheat were assessed by panelists for brownness, grain odor, sweet odor, grass odor, nutty taste, sweet taste, bitter taste, and taste (Figure 2).

Comparing the two brewing preparations, cold GWB received higher intensity scores for all sensory characteristics, especially color and odors. Among hot tea preparations, hot GWB_2 (2 g/100 mL) received the highest intensity scores for all characteristics, although it obtained similar scores as hot GWB_3 (4 g/100 mL) (Figure 2A). With respect to the appearance of hot GWB preparations, hot GWB_1 (0.8 g/100 mL) was lighter in color than hot GWB_2 (2 g/100 mL) and hot GWB_3 (4 g/100 mL), receiving the lowest brownness score (Figure 2C). However, hot GWB_2 (2 g/100 mL) received the highest consumer perception score. The intensities of the sensory characteristics for cold GWB prepared with different amounts of optimized roasted wheat are shown in Figure 2B,C. Panelists assigned higher intensity ratings for brownness, grain odor, grass odor, aftertaste, and bitter taste with increasing amount of roasted wheat powder in cold GWB preparations. However, the highest ratings for sweet taste, nutty taste, and sweet odor were received by cold GWB_2 (50 g/1000 mL). In addition, it can be assumed that consumers preferred cold brewing due to the stronger sensory intensities compared to hot infusion and a similar result was provided in the report of quality assessment of green tea on brewing conditions [39]. Upon the overall sensory evaluation, it was noteworthy that both types of germinated wheat beverages seemed to be acceptable as a new alternative due to its contemporary flavor and taste. However, the specific flavor profile related to its volatile components and nutritional profile could be explored for further understanding.

## 4. Conclusions

Recently, consumers looking for novel food products that could be easily consumed while maintaining and improving health. The grain-based beverages might be an effective way to meet consumer’s demand considering that germinated wheat contained higher bioactive compounds than commercial wheat. This study is the first report on possibility of GWB as grain-based beverage. In the results of this study, roasting time and temperature significantly affected the color characteristics, levels of bioactive compounds, and antioxidant capacity of germinated wheat. The formation of compounds related to the Maillard reaction was increased at higher temperatures and longer roasting times. Additionally, optimization of the roasting conditions enhanced the health-related properties of the germinated wheat. Process validation confirmed that the predicted response values concurred with experimental values under the optimized conditions. In addition, sensory evaluation revealed that the panelists preferred cold GWB to hot GWB prepared with the optimized roasted wheat powder, rating the intensity of the sensory characteristics higher for cold GWB than hot GWB. Therefore, the results of this study demonstrate that the appropriate roasting conditions for germinated wheat can enhance the bioactive content and antioxidant capacity of GWB while achieving acceptable sensory attributes and consumer acceptance.

## Figures and Tables

**Table 1:** Process variables, notation, and their actual values on coded levels.

Independent Variables	Notation	Coded Level				
		−*α*	−1	0	+1	+*α*
Temperature (°C)	X_1_	132	140	160	180	188
Time (min)	X_2_	1.7	10	30	50	58

X_n_: notation of independent variables. *α*: distance of axial point from the center.

**Table 2:** Experimental runs with actual values of independent variables.

Experiment No.	Actual Level	
	X_1_ Temperature (°C)	X_2_ Time (min)
1	140	10
2	180	10
3	140	50
4	180	50
5	132	30
6	188	30
7	160	1.7
8	160	58
9	160	30
10	160	30
11	160	30
12	160	30
13	160	30

X_n_: notation of independent variables.

**Table 3:** Experimental design used to CCD and experimental results of responses variables.

Experiment No.	Independent Variables		Response Variables					
	X_1_ Temperature (°C)	X_2_ Time (min)	Y_1_ BI ***	Y_2_ TFC *** (mg CE/g)	Y_3_ TPC *** (mg GE/g)	Y_4_ DPPH *** (µM TE/g)	Y_5_ TEAC *** (mM TE/g)	Y_6_ GABA *** (mg/g)
1	140 (−1)	10 (−1)	35.20 ± 1.24 ^h^	0.61 ± 0.01 ^cde^	0.94 ± 0.06 ^d^	1.30 ± 0.45 ^g^	3.94 ± 0.46 ^de^	1.37 ± 0.08 ^de^
2	180 (+1)	10 (−1)	57.24 ± 2.03 ^d^	0.62 ± 0.07 ^cde^	1.13 ± 0.07 ^cd^	2.98 ± 0.11 ^cd^	6.48 ± 0.36 ^de^	1.47 ± 0.03 ^de^
3	140 (−1)	50 (+1)	44.40 ± 2.11 ^f^	0.62 ± 0.03 ^cde^	0.94 ± 0.09 ^d^	1.95 ± 0.20 ^ef^	4.72 ± 0.07 ^de^	1.40 ± 0.10 ^de^
4	180 (+1)	50 (+1)	84.45 ± 0.77 ^a^	0.80 ± 0.04 ^a^	2.05 ± 0.10 ^a^	5.41 ± 0.50 ^a^	9.38 ± 0.11 ^a^	2.59 ± 0.09 ^a^
5	132 (−*α*)	30 (0)	42.15 ± 1.12 ^fg^	0.64 ± 0.07 ^cd^	0.94 ± 0.04 ^d^	2.02 ± 0.17 ^e^	4.77 ± 0.17 ^d^	1.56 ± 0.08 ^d^
6	188 (+*α*)	30 (0)	84.48 ± 1.80 ^a^	0.76 ± 0.06 ^ab^	2.04 ± 0.11 ^a^	4.96 ± 0.10 ^a^	9.76 ± 0.82 ^b^	2.08 ± 0.03 ^b^
7	160 (0)	1.7 (−*α*)	40.26 ± 1.54 ^g^	0.68 ± 0.08 ^bc^	0.93 ± 0.07 ^d^	1.48 ± 0.33 ^fg^	3.95 ± 0.01 ^de^	1.46 ± 0.15 ^de^
8	160 (0)	58 (+*α*)	63.53 ± 1.60 ^c^	0.60 ± 0.01 ^cde^	1.19 ± 0.08 ^c^	3.26 ± 0.13 ^bc^	6.83 ± 0.01 ^d^	1.55 ± 0.11 ^d^
9	160 (0)	30 (0)	67.44 ± 0.60 ^b^	0.56 ± 0.02 ^de^	1.44 ± 0.04 ^b^	3.52 ± 0.17 ^b^	7.53 ± 0.30 ^c^	1.87 ± 0.12 ^c^
10	160 (0)	30 (0)	58.31 ± 1.87 ^d^	0.59 ± 0.03 ^cde^	1.07 ± 0.09 ^cd^	2.98 ± 0.13 ^cd^	6.56 ± 0.47 ^f^	1.12 ± 0.12 ^f^
11	160 (0)	30 (0)	51.69 ± 1.40 ^e^	0.56 ± 0.05 ^de^	1.08 ± 0.10 ^cd^	2.63 ± 0.09 ^d^	6.27 ± 0.22 ^e^	1.33 ± 0.05 ^e^
12	160 (0)	30 (0)	51.99 ± 0.29 ^e^	0.65 ± 0.03 ^cd^	1.07 ± 0.09 ^cd^	2.75 ± 0.09 ^cd^	6.65 ± 0.52 ^ef^	1.27 ± 0.07 ^ef^
13	160 (0)	30 (0)	56.17 ± 1.03 ^d^	0.53 ± 0.02 ^e^	1.12 ± 0.12 ^cd^	2.92 ± 0.14 ^cd^	6.30 ± 0.16 ^ei^	1.34 ± 0.07 ^e^

Values are expressed means of three replications ± SD. Different superscript letters indicate a significant difference between samples. *** significantly differ at *p* < 0.001. X_n_: notation of independent variables; Y_n_: notation of response variables; CCD: Central Composite Design; BI: Browning Index, TFC: Total flavonoid content; TPC: Total phenolic content; DPPH: 2,2-diphenyl-1-picrylhydrazyl radical scavenging activity; TEAC: Trolox equivalent antioxidant capacity, GABA: gamma-amino butyric acid.

**Table 4:** Analysis of variance (ANOVA) for the response variables.

		BI	TFC	TPC	DPPH	TEAC	GABA
Constant	β_0_	56.72	0.5758	1.15	2.96	6.66	1.40
Linear	β_1_	15.24 ***	0.0459 *	0.3551 ***	1.16 ***	1.78 ***	0.2542 *
	β_2_	8.67 **	0.0096	0.1606 *	0.7014 ***	0.9684 ***	0.1611
Quadratic	β_11_	-	0.0592 *	0.1661 *	0.2594	0.2515	0.2188
	β_22_		0.0315	−0.0507	−0.3005 *	−0.6842 **	0.0615
Interaction	β_12_	-	0.0415	0.2313 *	0.4453 *	0.5277 *	0.2729
R^2^		0.8631	0.7306	0.9190	0.9637	0.9709	0.7499
Adjusted R^2^		0.8357	0.5381	0.8611	0.9377	0.9502	0.5712
Lack of Fit (*p* value)		0.5627	0.2638	0.6478	0.7288	0.9375	0.5529
*p* value		<0.0001	0.0554	0.0011	<0.0001	<0.0001	0.044

*, **, *** significantly differ at *p* < 0.05, *p* < 0.01, *p* < 0.001, respectively. β_0_: constant, β_1_: roasting temperature, β_2_: roasting time, R^2^: coefficient determination.

**Table 5:** Experimental runs with actual values of independent variables.

Responses	Goal	Predicted Value	Experimental Value	Absolute Residual Error (%)
BI	In range	78.73	73.54 ± 1.80	7.06
TFC (mg CE/g)	Maximize	0.74	0.72 ± 0.03	3.12
TPC (mg GE/g)	Maximize	1.95	1.96 ± 0.06	0.61
DPPH (μM TE/g)	Maximize	5.10	4.66 ± 0.18	9.41
TEAC (mM TE/g)	Maximize	9.45	10.20 ± 1.23	7.35
GABA (mg/g)	Maximize	2.25	2.46 ± 0.14	8.71

Values are expressed means of three replications ± SD.

**Table 6:** Consumer acceptability of germinated wheat beverages.

Samples	Appearance ***	Odor ***	Taste ***	Overall ***
Hot GWB_1	5.37 ± 1.37 ^b^	5.24 ± 1.78 ^c^	5.32 ± 1.71 ^c^	5.33 ± 1.77 ^b^
Hot GWB_2	5.60 ± 1.34 ^b^	4.97 ± 1.86 ^c^	5.45 ± 1.79 ^c^	5.49 ± 1.72 ^b^
Hot GWB_3	5.58 ± 1.40 ^b^	5.00 ± 1.78 ^c^	5.41 ± 1.63 ^c^	5.42 ± 1.60 ^b^
Cold GWB_1	6.30 ± 1.26 ^a^	6.06 ± 1.34 ^b^	6.32 ± 1.41 ^ab^	6.38 ± 1.33 ^a^
Cold GWB_2	6.52 ± 1.18 ^a^	6.54 ± 1.30 ^a^	6.62 ± 1.47 ^a^	6.54 ± 1.41 ^a^
Cold GWB_3	6.45 ± 1.35 ^a^	6.20 ± 1.38 ^ab^	5.99 ± 1.88 ^b^	6.13 ± 1.75 ^a^

GWB_1, Hot GWB_2, and Hot GWB_3 represent GWB prepared as hot beverages at different amounts of infusion (0.8, 2, and 4 g/100 mL), respectively. Cold GWB_1, Cold GWB_2, and Cold GWB_3 represent GWB prepared as cold beverages at different amounts of roasting germinated wheats (25, 50, and 75 g/1000 mL), respectively. *** significantly differ at *p* < 0.001. Different superscript letters indicate a significant difference between samples.

## Data Availability

Not applicable.
